# The genome sequence of the Mazarine Blue, *Cyaniris semiargus* (Rottemburg, 1775)

**DOI:** 10.12688/wellcomeopenres.19362.1

**Published:** 2023-04-24

**Authors:** Konrad Lohse, Alex Hayward, Dominik R. Laetsch, Valéria Marques, Roger Vila, Chris Tyler-Smith, Wellcome Sanger Institute Tree of Life programme, Wellcome Sanger Institute Scientific Operations: DNA Pipelines collective, Tree of Life Core Informatics collective, Darwin Tree of Life Consortium

**Affiliations:** 1 Institute of Ecology and Evolution, The University of Edinburgh, Edinburgh, Scotland, UK; 2 College of Life and Environmental Sciences, Department of Biosciences, University of Exeter, Exeter, England, UK; 3 Institut de Biologia Evolutiva (CSIC - Universitat Pompeu Fabra), Barcelona, Spain

**Keywords:** Cyaniris semiargus, Mazarine Blue, genome sequence, chromosomal, Lepidoptera

## Abstract

We present a genome assembly from an individual male *Cyaniris semiargus* (the Mazarine Blue; Arthropoda; Insecta; Lepidoptera; Lycaenidae). The genome sequence is 441.5 megabases in span. Most of the assembly is scaffolded into 24 chromosomal pseudomolecules, including the assembled Z sex chromosome. The mitochondrial genome has also been assembled and is 15.4 kilobases in length. Gene annotation of this assembly on Ensembl identified 16,408 protein coding genes.

## Species taxonomy

Eukaryota; Metazoa; Ecdysozoa; Arthropoda; Hexapoda; Insecta; Pterygota; Neoptera; Endopterygota; Lepidoptera; Glossata; Ditrysia; Papilionoidea; Lycaenidae; Polyommatinae; Polyommatini; Polyommatina; *Cyaniris*; *Cyaniris semiargus* (Rottemburg, 1775) (NCBI:txid 988025).

## Background

The Mazarine Blue, *Cyaniris semiargus* (Rottemburg, 1775), is a butterfly within the Lycaenidae family (the Blues, Coppers and Hairstreaks). Its English name comes from the blue-purple colour of the upper side of the male’s wings, ‘mazarine’ being derived either from Cardinal Mazarin (1602–1661, sometime first minister of France), who popularised a brilliant cut of diamonds that became known as ‘mazarines’ ( Bretherton, 1989) or alternatively his niece Hortense Mancini, Duchess Mazarin (1645–99) ( Marren, 2019). Like many ‘Blues’, the female’s wings are mostly brown, with blue basal scales that may extend notably in some specimens. In some populations, especially of the taxon *hellenus* Staudinger, 1862, from southern Greece, females display orange submarginal spots. The species has a Palearctic distribution, ranging from Morocco through much of Europe and Central Asia to China and Korea ( Tolman & Lewington, 1997).

The Mazarine Blue is listed as a species of Least Concern, with stable populations on a European assessment ( van Swaay *et al*., 2010), but with a decreasing trend on a Mediterranean assessment ( van Swaay *et al*., 2014) for the IUCN Red List. In Britain, the Mazarine Blue has been known as a rare indigenous species since 1710, but was last seen as a native around 1905 ( Andrews, 2015). It was formerly distributed widely in England and Wales, mainly in the south. The cause of extinction is suggested to be a change in agricultural practices ( Thomas & Lewington, 2010).

*Cyaniris semiargus* can be found in dry to damp meadows, grasslands, cultivations, forest clearings and margins, and mountain slopes up to 2,800 m, where the adults fly, usually as a single generation, between May and early August ( Haahtela, 2019). The larvae feed on various species of Fabaceae, including the genera *Trifolium*, *Melilotus*, *Coronilla*, *Vicia* and *Anthyllis*, and also on *Armeria* spp. ( Plumbaginaceae). In the southern part of its Palearctic distribution, this butterfly is restricted to somewhat isolated populations in mid to high altitude areas, except for restricted populations in south-western Iberia, where it inhabits coastal dunes and feeds exclusively on *Armeria velutina*. Again, like many other Blues, the larva and pupa have strong associations with ants of the genera *Lasius* and *Camponotus*, which obtain a sweet secretion from the larva and provide protection ( Thomas & Lewington, 2010).

The genus *Cyaniris* seems to be closely related to *Rimisia* and *Agriades*, with a divergence of about 4.4 Myr ( Talavera *et al.*, 2013). The Mazarine Blue has a haploid chromosome number of 24 ( de Lesse, 1960; Federley, 1938; Lorković, 1941). The genome sequence now provides the basis for understanding the genetic relationships of the Mazarine Blue to other Lycaenidae and for studies on sexual dimorphism, myrmecophily and ecological adaptation. It will also help make sense of the genetic diversity and phylogeography of this species and its multiple recorded subspecies.

### Genome sequence report

**Figure 1.**
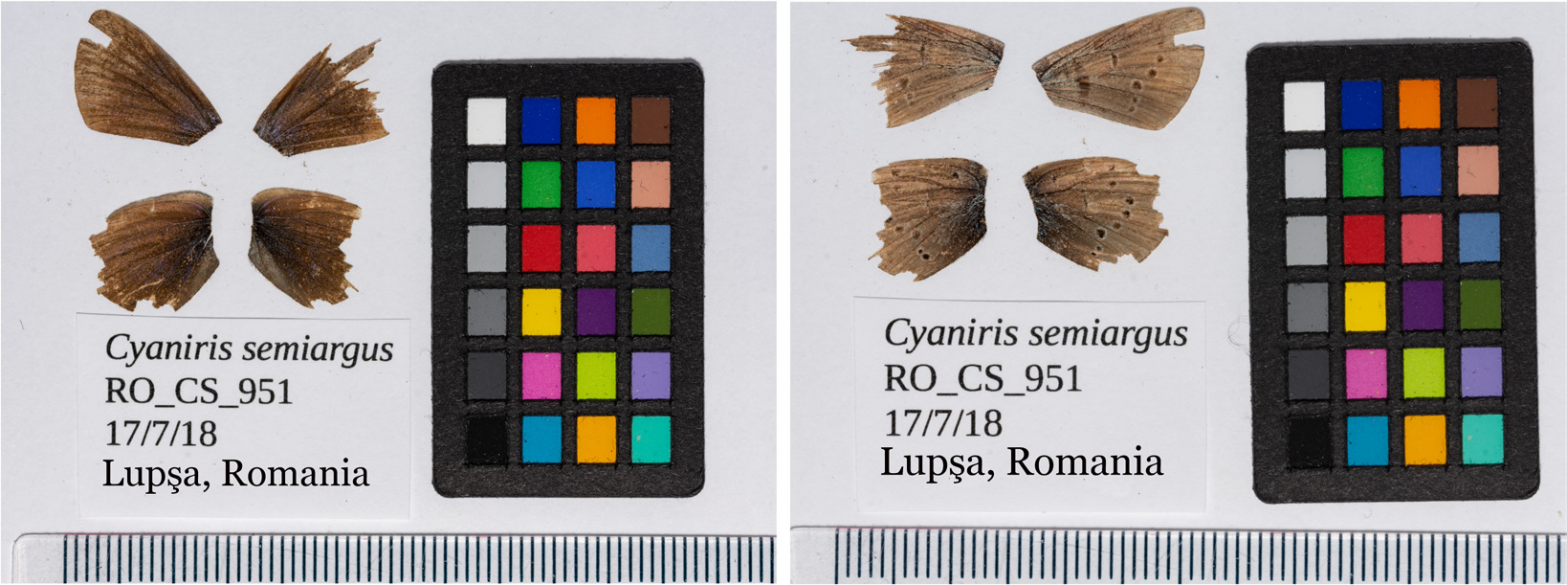
Forewings and hindwings of the male *Cyaniris semiargus* (ilCyaSemi1) specimen from which the genome was sequenced. Dorsal ( **a**) and ventral ( **b**) surface view of wings from specimen RO_CS_951 from Lupşa, Alba, Romania, used to generate Pacific Biosciences, 10X genomics and Hi-C data.

The genome was sequenced from one male *Cyaniris semiargus* specimen ( Figure 1) collected from Apuseni Mountains, Lupșa, Alba, Romania (latitude 46.42, longitude 23.19). A total of 42-fold coverage in Pacific Biosciences single-molecule HiFi long reads and 77-fold coverage in 10X Genomics read clouds were generated. Primary assembly contigs were scaffolded with chromosome conformation Hi-C data. Manual assembly curation corrected five missing joins or mis-joins, reducing the scaffold number by 10.71%.

**Figure 2.**
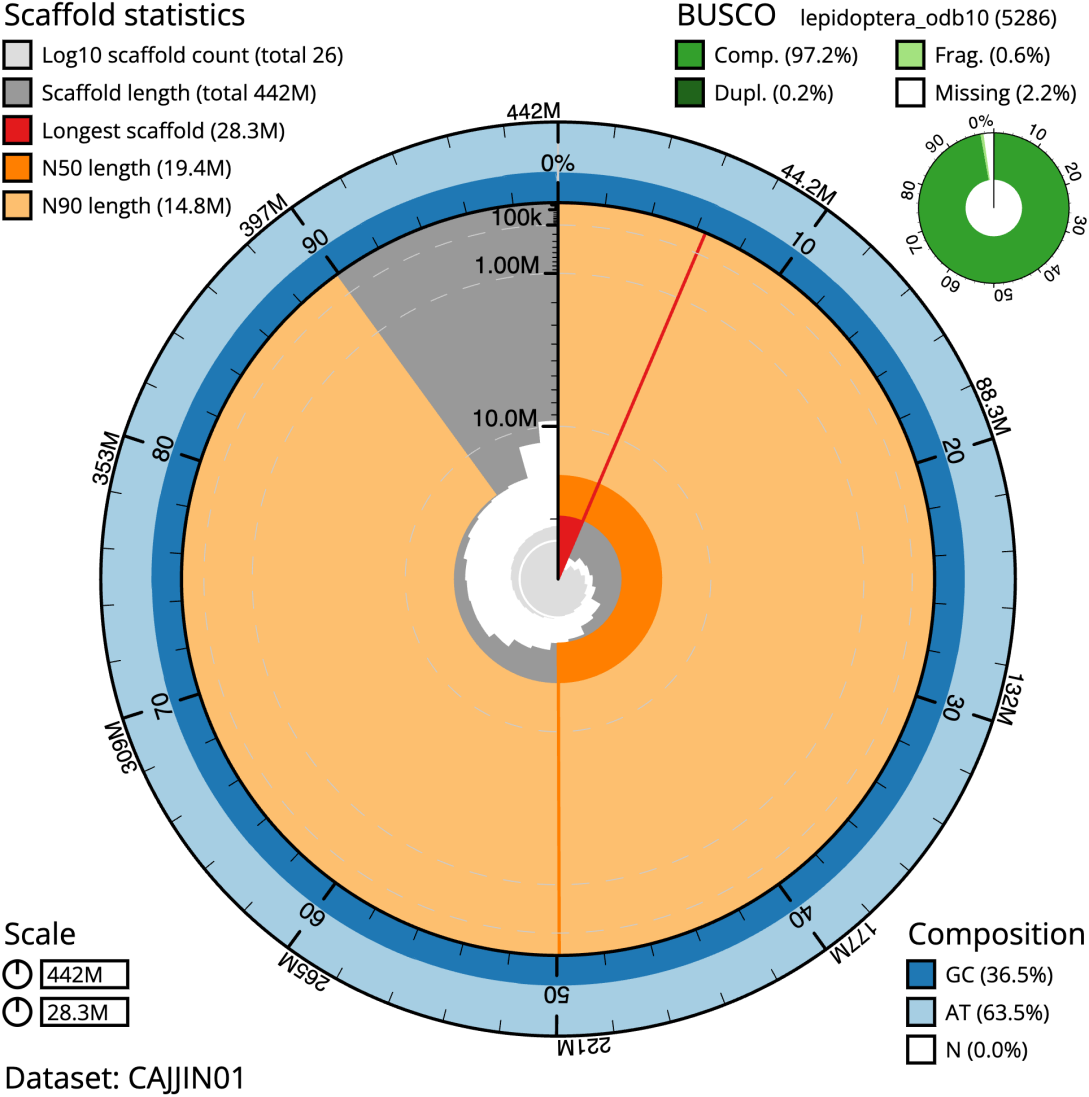
Genome assembly of *Cyaniris semiargus*, ilCyaSemi1.1: metrics. The BlobToolKit Snailplot shows N50 metrics and BUSCO gene completeness. The main plot is divided into 1,000 size-ordered bins around the circumference with each bin representing 0.1% of the 441,519,306 bp assembly. The distribution of scaffold lengths is shown in dark grey with the plot radius scaled to the longest scaffold present in the assembly (28,312,907 bp, shown in red). Orange and pale-orange arcs show the N50 and N90 scaffold lengths (19,361,588 and 14,815,647 bp), respectively. The pale grey spiral shows the cumulative scaffold count on a log scale with white scale lines showing successive orders of magnitude. The blue and pale-blue area around the outside of the plot shows the distribution of GC, AT and N percentages in the same bins as the inner plot. A summary of complete, fragmented, duplicated and missing BUSCO genes in the lepidoptera_odb10 set is shown in the top right. An interactive version of this figure is available at https://blobtoolkit.genomehubs.org/view/ilCyaSemi1.1/dataset/CAJJIN01/snail#Filters.

The final assembly has a total length of 441.5 Mb in 25 sequence scaffolds with a scaffold N50 of 19.4 Mb ( Table 1). Most (99.99%) of the assembly sequence was assigned to 24 chromosomal-level scaffolds, representing 23 autosomes, and the Z sex chromosome. Chromosome-scale scaffolds confirmed by the Hi-C data are named in order of size ( Figure 2– Figure 5; Table 2). While not fully phased, the assembly deposited is of one haplotype. Contigs corresponding to the second haplotype have also been deposited.

**Table 1.** Genome data for *Cyaniris semiargus*, ilCyaSemi1.1.

Project accession data		
Assembly identifier	ilCyaSemi1.1	
Species	*Cyaniris semiargus*	
Specimen	ilCyaSemi1	
NCBI taxonomy ID	988025	
BioProject	PRJEB42123	
BioSample ID	SAMEA7523311	
Isolate information	ilCyaSemi1, whole organism (genome sequencing, Hi-C scaffolding)	
Assembly metrics *		*Benchmark*
Consensus quality (QV)	56	*≥ 50*
*k*-mer completeness	99.99%	*≥ 95%*
BUSCO **	C:97.2%[S:96.9%,D:0.2%], F:0.6%,M:2.2%,n:5,286	*C ≥ 95%*
Percentage of assembly mapped to chromosomes	99.99%	*≥ 95%*
Sex chromosomes	Z chromosome	*localised homologous pairs*
Organelles	Mitochondrial genome assembled.	*complete single alleles*
Raw data accessions		
PacificBiosciences SEQUEL II	ERR6560795	
10X Genomics Illumina	ERR6002620–ERR6002623	
Hi-C Illumina	ERR6002624–ERR6002626, ERR6003039	
Genome assembly		
Assembly accession	GCA_905187585.1	
*Accession of alternate haplotype*	GCA_905147265.1	
Span (Mb)	441.5	
Number of contigs	34	
Contig N50 length (Mb)	16.3	
Number of scaffolds	25	
Scaffold N50 length (Mb)	19.4	
Longest scaffold (Mb)	28.3	
Genome annotation		
Number of protein-coding genes	16,408	
Number of gene transcripts	16,601	

* Assembly metric benchmarks are adapted from column VGP-2020 of “Table 1: Proposed standards and metrics for defining genome assembly quality” from ( Rhie *et al.*, 2021). ** BUSCO scores based on the lepidoptera_odb10 BUSCO set using v5.3.2. C = complete [S = single copy, D = duplicated], F = fragmented, M = missing, n = number of orthologues in comparison. A full set of BUSCO scores is available at https://blobtoolkit.genomehubs.org/view/ilCyaSemi1.1/dataset/CAJJIN01/busco.

**Figure 3.**
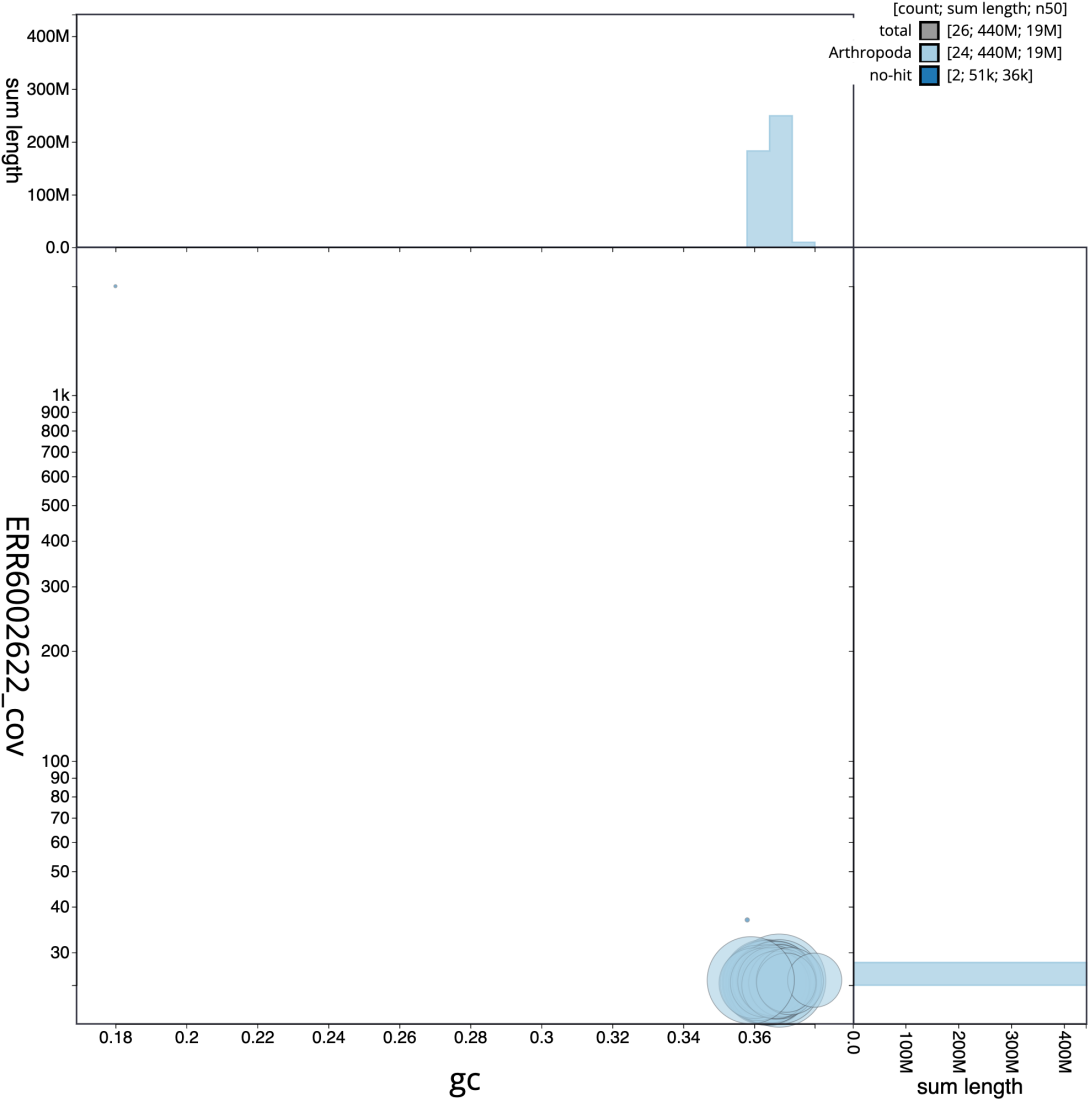
Genome assembly of *Cyaniris semiargus*, ilCyaSemi1.1: BlobToolKit GC-coverage plot. Scaffolds are coloured by phylum. Circles are sized in proportion to scaffold length. Histograms show the distribution of scaffold length sum along each axis. An interactive version of this figure is available at https://blobtoolkit.genomehubs.org/view/ilCyaSemi1.1/dataset/CAJJIN01/blob.

**Figure 4.**
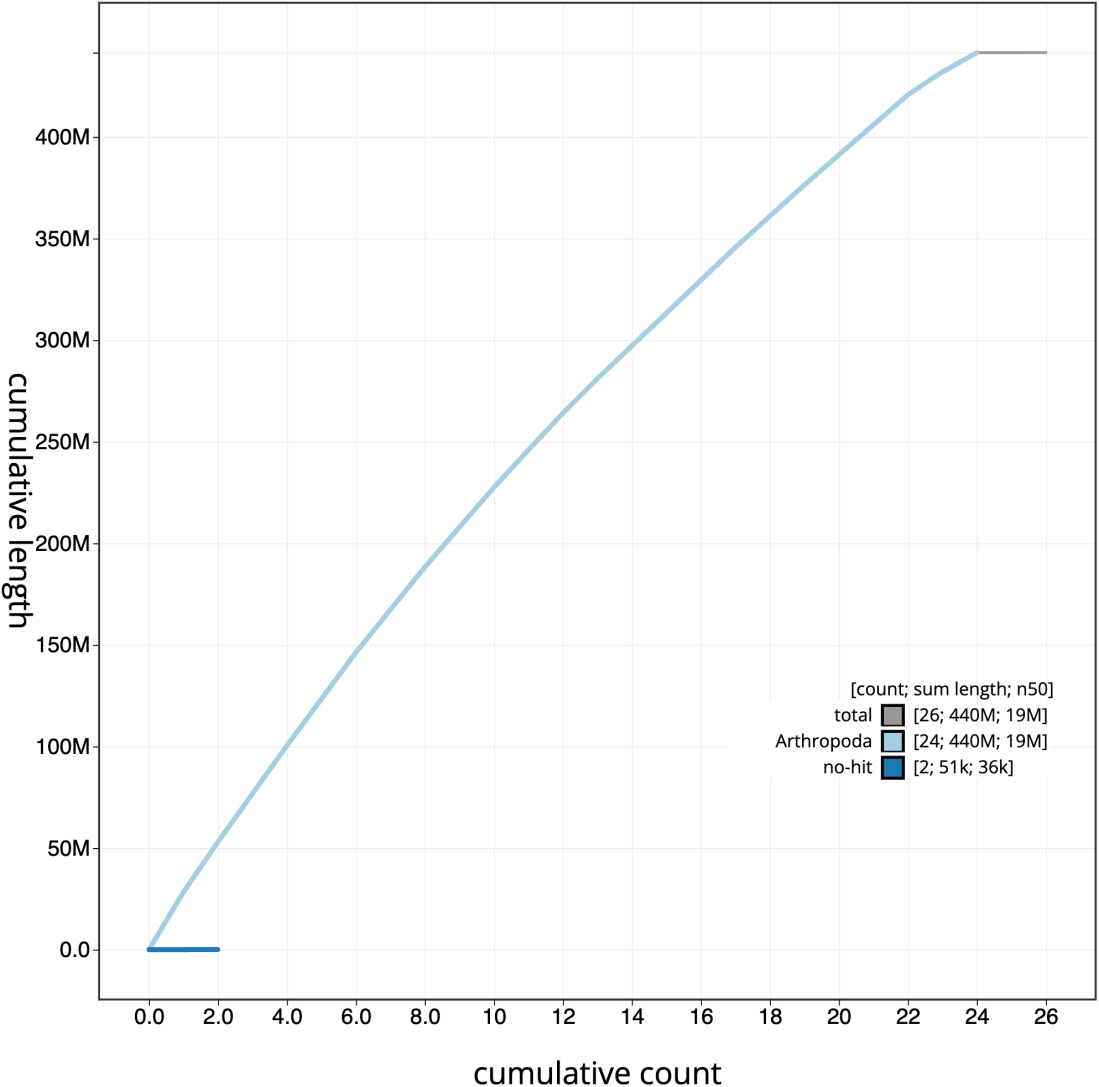
Genome assembly of *Cyaniris semiargus*, ilCyaSemi1.1: BlobToolKit cumulative sequence plot. The grey line shows cumulative length for all scaffolds. Coloured lines show cumulative lengths of scaffolds assigned to each phylum using the buscogenes taxrule. An interactive version of this figure is available at https://blobtoolkit.genomehubs.org/view/ilCyaSemi1.1/dataset/CAJJIN01/cumulative.

**Figure 5.**
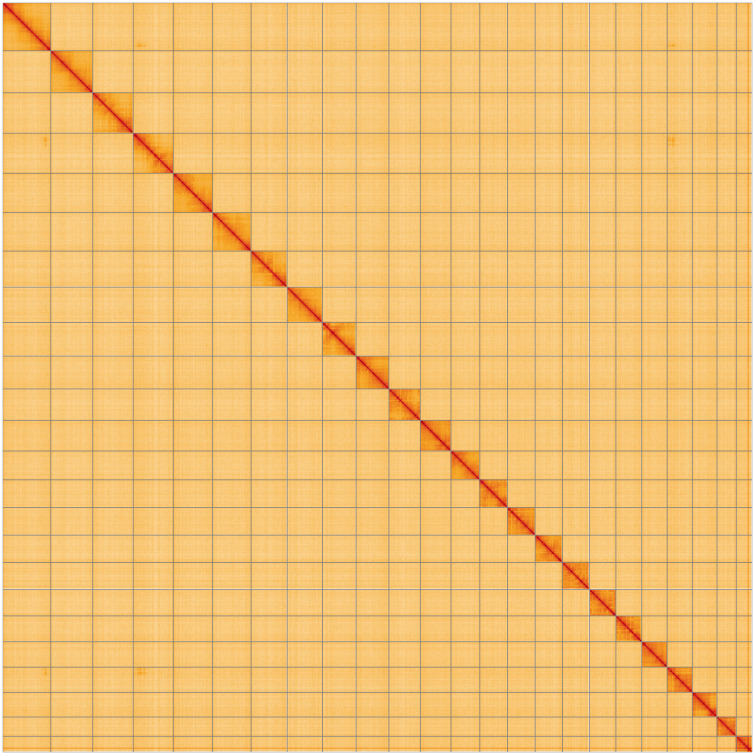
Genome assembly of *Cyaniris semiargus*, ilCyaSemi1.1: Hi-C contact map of the ilCyaSemi1.1 assembly, visualised using HiGlass. Chromosomes are shown in order of size from left to right and top to bottom. An interactive version of this figure may be viewed at https://genome-note-higlass.tol.sanger.ac.uk/l/?d=RaIZ0Z3sQmercnKuDiEVdQ.

**Table 2.** Chromosomal pseudomolecules in the genome assembly of *Cyaniris semiargus*, ilCyaSemi1.

INSDC accession	Chromosome	Size (Mb)	GC%
LR994546.1	1	28.31	36.7
LR994548.1	2	23.83	36.3
LR994549.1	3	23.59	36.4
LR994550.1	4	23.19	36.7
LR994551.1	5	22.67	36.3
LR994552.1	6	21.24	36.5
LR994553.1	7	20.83	36.4
LR994554.1	8	19.76	36.6
LR994555.1	9	19.36	36.6
LR994556.1	10	18.47	36.1
LR994557.1	11	18.09	36.7
LR994558.1	12	17	36.4
LR994559.1	13	16.28	36.7
LR994560.1	14	16.26	36.4
LR994561.1	15	16.13	36
LR994562.1	16	15.95	36.6
LR994563.1	17	15.52	36.3
LR994564.1	18	15.2	36.5
LR994565.1	19	14.97	36.8
LR994566.1	20	14.82	36.6
LR994567.1	21	14.58	37
LR994568.1	22	11.32	36.9
LR994569.1	23	9.41	37.7
LR994547.1	Z	24.71	35.9
LR994570.1	MT	0.02	18.3
-	unplaced	0.04	35.8

The estimated *k*-mer-based Quality Value (QV) of the final assembly is 56.0 with *k*-mer based completeness of 99.99%, and the assembly has a BUSCO v5.3.2 completeness of 97.2% (single = 96.9%, duplicated = 0.2%), using the lepidoptera_odb10 reference set ( *n* = 5,286).

Metadata for specimens, spectral estimates, sequencing runs, contaminants and pre-curation assembly statistics can be found here.

### Genome annotation report

The *Cyaniris semiargus* genome assembly GCA_905187585.1 (ilCyaSemi1.1) genome was annotated using the Ensembl rapid annotation pipeline ( Table 1). The resulting annotation includes 16,601 transcribed mRNAs from 16,408 protein-coding genes.

## Methods

### Sample acquisition and nucleic acid extraction

A male *Cyaniris semiargus* specimen (specimen number RO-CS-951, ToLID ilCyaSemi1) was collected from Apuseni Mountains, Lupșa, Alba, Romania (latitude 46.42, longitude 23.19) on 17 July 2018 using a hand net. The members of the collection team were Konrad Lohse (University of Edinburgh), Alex Hayward (University of Exeter), Dominik Laetsch (University of Edinburgh), and Roger Vila (Institut de Biologia Evolutiva). The specimen was identified by Roger Vila and then snap-frozen from live in a dry shipper.

DNA was extracted at the Tree of Life laboratory, Wellcome Sanger Institute (WSI). The ilCyaSemi1 sample was weighed and dissected on dry ice with tissue set aside for Hi-C sequencing. Whole organism tissue was disrupted using a Nippi Powermasher fitted with a BioMasher pestle. High molecular weight (HMW) DNA was extracted using the Qiagen MagAttract HMW DNA extraction kit. Low molecular weight DNA was removed from a 20-ng aliquot of extracted DNA using the 0.8X AMpure XP purification kit prior to 10X Chromium sequencing; a minimum of 50 ng DNA was submitted for 10X sequencing. HMW DNA was sheared into an average fragment size of 12–20 kb in a Megaruptor 3 system with speed setting 30. Sheared DNA was purified by solid-phase reversible immobilisation using AMPure PB beads with a 1.8X ratio of beads to sample to remove the shorter fragments and concentrate the DNA sample. The concentration of the sheared and purified DNA was assessed using a Nanodrop spectrophotometer and Qubit Fluorometer and Qubit dsDNA High Sensitivity Assay kit. Fragment size distribution was evaluated by running the sample on the FemtoPulse system.

### Sequencing

Pacific Biosciences HiFi circular consensus and 10X Genomics read cloud DNA sequencing libraries were constructed according to the manufacturers’ instructions. DNA sequencing was performed by the Scientific Operations core at the WSI on Pacific Biosciences SEQUEL II (HiFi) and HiSeq X Ten (10X) instruments. Hi-C data were also generated from tissue of ilCyaSemi1 using the Arima v1 kit and sequenced on the HiSeq X Ten instrument.

### Genome assembly, curation and evaluation

Assembly was carried out with Hifiasm ( Cheng *et al.*, 2021) and haplotypic duplication was identified and removed with purge_dups ( Guan *et al.*, 2020). One round of polishing was performed by aligning 10X Genomics read data to the assembly with Long Ranger ALIGN, calling variants with FreeBayes ( Garrison & Marth, 2012). The assembly was then scaffolded with Hi-C data ( Rao *et al.*, 2014) using SALSA2 ( Ghurye *et al.*, 2019). The assembly was checked for contamination and corrected using the gEVAL system ( Chow *et al.*, 2016) as described previously ( Howe *et al.*, 2021). Manual curation was performed using gEVAL, HiGlass ( Kerpedjiev *et al.*, 2018) and Pretext ( Harry, 2022). The mitochondrial genome was assembled using MitoHiFi ( Uliano-Silva *et al.*, 2022), which runs MitoFinder ( Allio *et al.*, 2020) or MITOS ( Bernt *et al.*, 2013) and uses these annotations to select the final mitochondrial contig and to ensure the general quality of the sequence. To evaluate the assembly, MerquryFK was used to estimate consensus quality (QV) scores and *k*-mer completeness ( Rhie *et al.*, 2020). The genome was analysed within the BlobToolKit environment ( Challis *et al.*, 2020) and BUSCO scores ( Manni *et al.*, 2021; Simão *et al.*, 2015) were calculated. Table 3 contains a list of software tool versions and sources.

**Table 3.** Software tools: versions and sources.

Software tool	Version	Source
BlobToolKit	4.0.7	https://github.com/blobtoolkit/blobtoolkit
BUSCO	5.3.2	https://gitlab.com/ezlab/busco
FreeBayes	1.3.1-17-gaa2ace8	https://github.com/freebayes/freebayes
gEVAL	N/A	https://geval.org.uk/
Hifiasm	0.12	https://github.com/chhylp123/hifiasm
HiGlass	1.11.6	https://github.com/higlass/higlass
Long Ranger ALIGN	2.2.2	https://support.10xgenomics.com/genome-exome/ software/pipelines/latest/advanced/other-pipelines
MitoHiFi	1	https://github.com/marcelauliano/MitoHiFi
PretextView	0.2	https://github.com/wtsi-hpag/PretextView
purge_dups	1.2.3	https://github.com/dfguan/purge_dups
SALSA	2.2	https://github.com/salsa-rs/salsa

### Genome annotation

The BRAKER2 pipeline ( Brůna *et al.*, 2021) was used in the default protein mode to generate annotation for the *Cyaniris semiargus* assembly (GCA_905187585.1) in Ensembl Rapid Release.

### Ethics and compliance issues

The materials that have contributed to this genome note have been supplied by a Darwin Tree of Life Partner. The submission of materials by a Darwin Tree of Life Partner is subject to the Darwin Tree of Life Project Sampling Code of Practice. By agreeing with and signing up to the Sampling Code of Practice, the Darwin Tree of Life Partner agrees they will meet the legal and ethical requirements and standards set out within this document in respect of all samples acquired for, and supplied to, the Darwin Tree of Life Project. All efforts are undertaken to minimise the suffering of animals used for sequencing. Each transfer of samples is further undertaken according to a Research Collaboration Agreement or Material Transfer Agreement entered into by the Darwin Tree of Life Partner, Genome Research Limited (operating as the Wellcome Sanger Institute), and in some circumstances other Darwin Tree of Life collaborators.

## Data Availability

European Nucleotide Archive: *Cyaniris semiargus* (mazarine blue). Accession number PRJEB42123; https://identifiers.org/ena.embl/PRJEB42123 ( Wellcome Sanger Institute, 2020) The genome sequence is released openly for reuse. The *Cyaniris semiargus* genome sequencing initiative is part of the Darwin Tree of Life (DToL) project. All raw sequence data and the assembly have been deposited in INSDC databases. Raw data and assembly accession identifiers are reported in Table 1.

## Author information

Members of the Wellcome Sanger Institute Tree of Life programme are listed here: https://doi.org/10.5281/zenodo.4783585.

Members of Wellcome Sanger Institute Scientific Operations: DNA Pipelines collective are listed here: https://doi.org/10.5281/zenodo.4790455.

Members of the Tree of Life Core Informatics collective are listed here: https://doi.org/10.5281/zenodo.5013541.

Members of the Darwin Tree of Life Consortium are listed here: https://doi.org/10.5281/zenodo.4783558.
