# Supplementary material for: The genome sequence of the Mazarine Blue, Cyaniris semiargus (Rottemburg, 1775)
Source: Wellcome Open Res. 2023 Apr 24;8:181. [Version 1] doi: 10.12688/wellcomeopenres.19362.1 (PMC11109581; doi:10.12688/wellcomeopenres.19362.1)
Supplement: Peer review history

# Reviewer report: Reviewer response for version 1

**DOI:** 10.21956/wellcomeopenres.21447.r80601

**Published:** 2024-05-13

**Authors:** Mario Alejandro Marín

**Affiliations:** 1 Animal Universidade Estadual de Campinas, Antioquia, Colombia

The current work is relevant and of general interest to researchers interested in genomic assembly, butterfly evolution, and Lepidoptera. It provides relevant information about Palearctic Lycaenidae. 

 The studied butterfly, *C. semiargus, is declining* in the Mediterranean region and is extinct locally in Britain. An annotated genome of this species would be useful for its conservation. 

 The manuscript clearly describes the methodology, including the butterfly collection, laboratory procedures, genome assembly, and annotation. Unfortunately, the butterfly wings were deteriorated during the collection process (Figure 1). For further genomic studies, I encourage the researchers to manipulate the specimens more carefully. Additionally, it is suggested that these wings be mounted on a plate and included in a natural history collection.   

 The manuscript retains some mistakes mentioned by the previous reviewer, such as the author's affiliation, Tyler-Smith, and the link to the interactive version of Figure 5.

Are sufficient details of methods and materials provided to allow replication by others?

Yes

Is the rationale for creating the dataset(s) clearly described?

Yes

Are the datasets clearly presented in a useable and accessible format?

Yes

Are the protocols appropriate and is the work technically sound?

Yes

Reviewer Expertise:

Butterfly's systematic and ecology

I confirm that I have read this submission and believe that I have an appropriate level of expertise to confirm that it is of an acceptable scientific standard.

# Reviewer report: Reviewer response for version 1

**DOI:** 10.21956/wellcomeopenres.21447.r56491

**Published:** 2023-05-09

**Authors:** William Walker III

**Affiliations:** 1 Temperate Tree Fruit and Vegetable Research Unit, USDA-ARS United States National Arboretum, Washington, District of Columbia, USA

The authors present a chromosomally compete genome sequence assembly for the Mazarine Blue butterfly, *Cyaniris semiargus*. The genome is sequenced from a single male, with a total of 42-fold coverage from HiFi long reads, 77-fold coverage from 10X Genomics read clouds and chromosome confirmation Hi-C data. Manual assembly curations are performed to improve the assembly. Mitochondrial genome assembly is also provided. Genome annotation predictions are made for number of protein coding genes and number of gene transcripts. Standard bioinformatics pipelines are followed, with all software clearly indicated in Table 3.

 For genome annotation predictions, it is not clear if any RNA-Seq/transcriptome data was utilized to facilitate or improve gene annotation predictions.

 In Figure 1, it is not clear what the scale of the ruler is at the bottom of each panel of each panel, nor what the relevance of the color palette is. Some further information in the figure legend would be appreciated if these items are to be included.

 In Figure 3, on the X axis, GC% is indicated, but it is not clear what is being shown on the Y axis. The apparent accession is indicated, ERR6002622_cov, but it is not clear what this is referring to, nor what are the units of the metrics being shown for this axis.

 In the Data Availability section, the link to accession PRJEB42123 seems to not be working. The following error message is indicated. “Error: 500. There was a technical error. Something has gone wrong with our web server when attempting to make this page. Unfortunately, the service you are trying to access is currently unavailable. Please try again later.” The scope of this error is uncertain but the link was not working just prior to submitting this review.

 Please check the affiliation attributions for all the authors. The superscript for author Tyler-Smith is indicated as “0”, but there is no institute attributed to “0” below.

 Finally, a cosmetic note concerning Figure 5, it may be useful to the reader if Chromosome labels are indicated, at least for the Z chromosomes as this is specifically mentioned in the abstract, and there are otherwise no clear indications in the figure which plots correlate specifically to these chromosomes. (This is mentioned because in another similar Data Note in this journal, there is an a similar Hi-C contact plot with an online interactive version, in which the chromosome accessions are clearly shown for each box). Furthermore, the link to the interactive version of this Figure, found in the figure legend, does not seem to work. The HiGlass website opens up, but the figure never shows up and seems to be “Loading” indefinitely. It was attempted to access the link with different web-browsers, with no luck, and there were no issues accessing the other interactive online figures.

Are sufficient details of methods and materials provided to allow replication by others?

Yes

Is the rationale for creating the dataset(s) clearly described?

Yes

Are the datasets clearly presented in a useable and accessible format?

Partly

Are the protocols appropriate and is the work technically sound?

Yes

Reviewer Expertise:

Insect genetics, genomics/transcriptomics, and molecular biology.

I confirm that I have read this submission and believe that I have an appropriate level of expertise to confirm that it is of an acceptable scientific standard.
